# PB.02: Magnetic resonance imaging axilla: friend or foe?

**DOI:** 10.1186/bcr3504

**Published:** 2013-11-08

**Authors:** CF Hathaway, C Zammit, S Shaheed, G Rubin, J O'Brien

**Affiliations:** 1 Brighton and Sussex Medical School, Brighton, UK; 2 Department of Surgery, Park Centre for Breast Care, Brighton, UK; 3 Department of Radiology, Park Centre for Breast Care, Brighton, UK

## Introduction

Breast MRI is used for local staging of breast cancer. It also gives information on the axilla. We looked at patients whose staging breast MRI revealed indeterminate axillary node appearances.

## Methods

A retrospective analysis was conducted of all breast MRIs in one institution between July 2012 and June 2013. MRI scans that found indeterminate axillary nodes and recommended second-look ultrasound were analysed, and correlated with fine-needle aspiration (FNA) cytology and surgical histology results.

## Results

A total of 180 MRI scans were performed for local breast cancer staging, having met NICE early cancer guidelines. Ten had incomplete follow up, leaving 170 in the study. In total, 131 correlated with the primary axillary ultrasound imaging and cytology, and did not have further axillary investigation. Thirty-nine (22.9%) requested second-look ultrasounds, with 25 requiring FNA; 10 (5.9%) found positive, 15 (8.8%) found negative nodes. Nodes found to be positive on FNA had axillary clearance rather than sentinel node biopsy, confirmed on surgical histology in all 10. Of the 14 patients who did not have an FNA on second look, only one was found to be positive on later histology. Two of the 15 had FNA on second look, and given a negative result, did ultimately have positive nodes on sentinel node biopsy.

## Conclusion

MRI and second-look ultrasound/FNA correctly identified 10 (5.9%) more patients with involved nodes than initial staging. Twenty-nine (17.1%) other patients had additional axillary ultrasound including FNA in 15 (8.8%) without a change in axillary management.

